# Vagusnervstimulation bei schwer zu behandelnden Depressionen

**DOI:** 10.1007/s00115-022-01282-6

**Published:** 2022-04-05

**Authors:** C. Reif-Leonhard, A. Reif, B. T. Baune, E. Kavakbasi

**Affiliations:** 1 Klinik für Psychiatrie, Psychosomatik und Psychotherapie, Universitätsklinikum Frankfurt – Goethe-Universität, Heinrich-Hoffmann-Straße 10, 60528 Frankfurt am Main, Deutschland; 2 Klinik für Psychische Gesundheit, Universitätsklinikum Münster – Westfälische Wilhelms-Universität Münster, Münster, Deutschland; 3 Department of Psychiatry, Melbourne Medical School, The University of Melbourne, Melbourne, Australien; 4 The Florey Institute of Neuroscience and Mental Health, The University of Melbourne, Melbourne, Australien

**Keywords:** Neuromodulatorisches Netzwerk, Wirkmechanismus, Langzeiteffekt, Adjuvantes Verfahren, Neurostimulation, Neuromodulatory network, Mechanism of action, Long-term effect, Adjuvant procedure, Neurostimulation

## Abstract

**Einführung:**

Seit 20 Jahren ist die Vagusnervstimulation (VNS) eine europaweit zugelassene invasive Therapieoption für therapieresistente Depressionen (TRD). Im Gegensatz zu geläufigeren Behandlungen wie EKT sind Kenntnisse über VNS sowohl in der Allgemeinbevölkerung als auch in Fachkreisen gering.

**Methoden:**

In diesem narrativen Review geben wir eine klinisch und wissenschaftlich fundierte Übersicht über die VNS. Hypothesen zum Wirkmechanismus sowie die aktuelle Evidenzlage zur Wirksamkeit werden dargestellt. Das perioperative Management, das Nebenwirkungsprofil und die Nachbetreuung einschließlich Dosistitration werden beschrieben. Ein Vergleich über internationale Leitlinienempfehlungen zur VNS findet sich ebenfalls. Ferner formulieren wir Kriterien, die bei der Auswahl geeigneter Patienten hilfreich sind.

**Ergebnisse:**

Die elektrischen Impulse werden über den N. vagus afferent weitergeleitet und stimulieren über verschiedene Wege ein neuromodulatorisches zerebrales Netzwerk. Viele Studien und Fallserien zeigten die Wirksamkeit von VNS als adjuvantes Verfahren bei TRD. Der Effekt tritt mit einer Latenz von 3 bis 12 Monaten ein und steigt möglicherweise mit der Dauer der VNS. Unter der Beachtung der Stimulationsempfehlungen sind die Nebenwirkungen für die meisten Patienten tolerabel.

**Fazit:**

Die VNS ist eine zugelassene, wirksame und gut verträgliche Langzeittherapie für chronische und therapieresistente Depressionen. Weitere Sham-kontrollierte Studien über einen längeren Beobachtungszeitraum sind zur Verbesserung der Evidenz wünschenswert.

**Zusatzmaterial online:**

Die Online-Version dieses Beitrags (10.1007/s00115-022-01282-6) enthält eine weitere Infobox. Beitrag und Zusatzmaterial stehen Ihnen auf www.springermedizin.de zur Verfügung. Bitte geben Sie dort den Beitragstitel in die Suche ein, das Zusatzmaterial finden Sie beim Beitrag unter „Ergänzende Inhalte“.

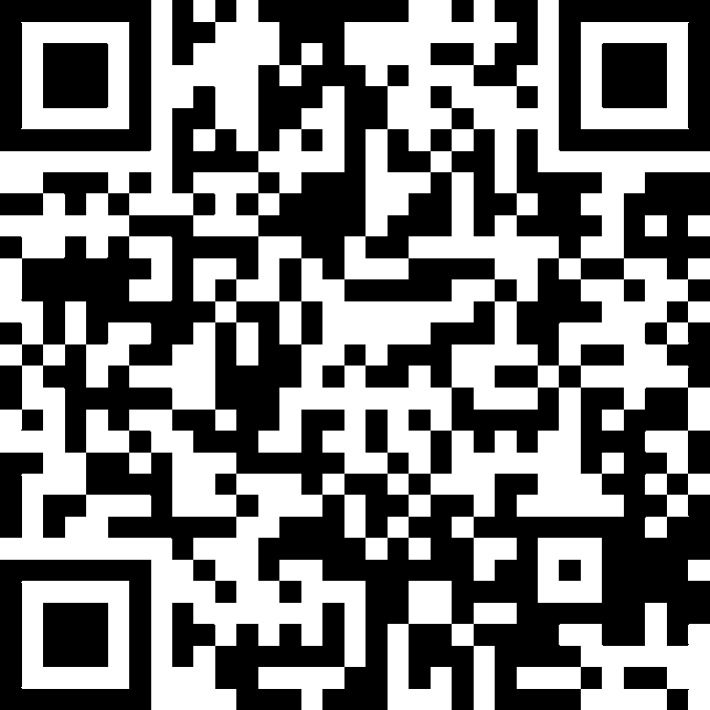

## Abstract (en)

**Introduction:**

For the past 20 years vagus nerve stimulation (VNS) has been an approved invasive treatment option for treatment-resistant depression (TRD) across Europe. In contrast to more common treatments, such as ECT, knowledge about VNS is low both in the general population and among professionals.

**Methods:**

In this narrative review, we provide a clinically and scientifically sound overview of VNS. Hypotheses on the mechanism of action as well as the current evidence base on efficacy are presented. Perioperative management, adverse event profile and follow-up including dose titration are described. A comparison of international guideline recommendations on VNS is also provided. Furthermore, we formulate criteria that are helpful in the selection of appropriate patients.

**Results:**

Electrical impulses are transmitted afferently via the vagus nerve and stimulate a neuromodulatory cerebral network via different pathways. Many studies and case series demonstrated the efficacy of VNS as an adjuvant procedure for TRD. The effect occurs with a latency period of 3–12 months and possibly increases with the duration of VNS. If stimulation recommendations are followed, side effects are tolerable for most patients.

**Conclusion:**

The use of VNS is an approved, effective and well-tolerated long-term therapy for chronic and treatment-resistant depression. Further sham-controlled studies over a longer observational period are desirable to improve the evidence.

Seit 2001 ist die Vagusnervstimulation (VNS) in der Behandlung therapieresistenter Depressionen (TRD) in Europa zugelassen. In Deutschland werden die Kosten für den minimal-invasiven Eingriff von den Krankenkassen übernommen. Während die Behandlung in den ersten Jahren zunächst vorwiegend an einigen wenigen universitären Zentren angeboten wurde, entstanden in den letzten 5 Jahren deutschlandweit über 30 VNS-Spezialambulanzen. Aufgrund dessen sollen in dieser Übersichtsarbeit zum einen die aktuelle Datenlage zur Wirksamkeit und zu Hypothesen zum Wirkmechanismus dargestellt sowie praktische Empfehlungen zur Patientenauswahl und Einrichtung einer VNS-Spezialambulanz auf der Basis umfangreicher eigener Erfahrungen formuliert werden.

## Grundlagen der VNS und Wirkweise

**Figure 1:**
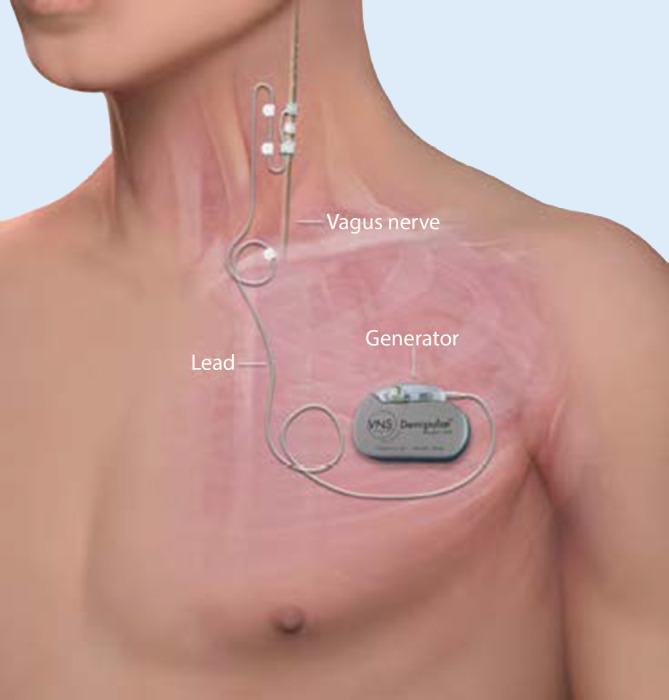

**Figure 2:**
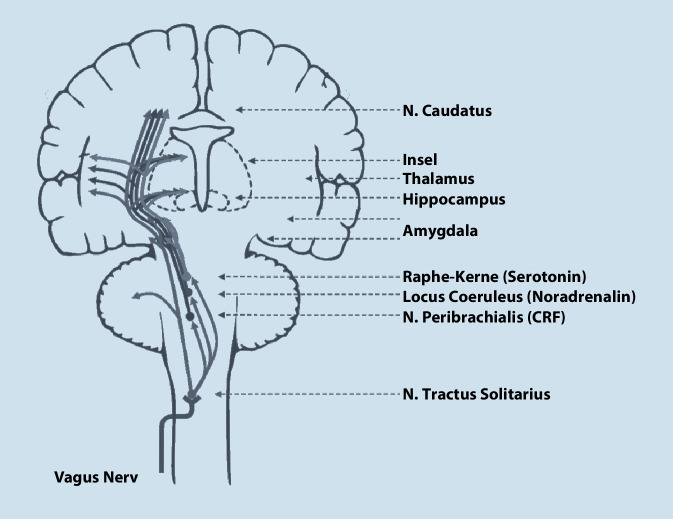

**Figure 3:**
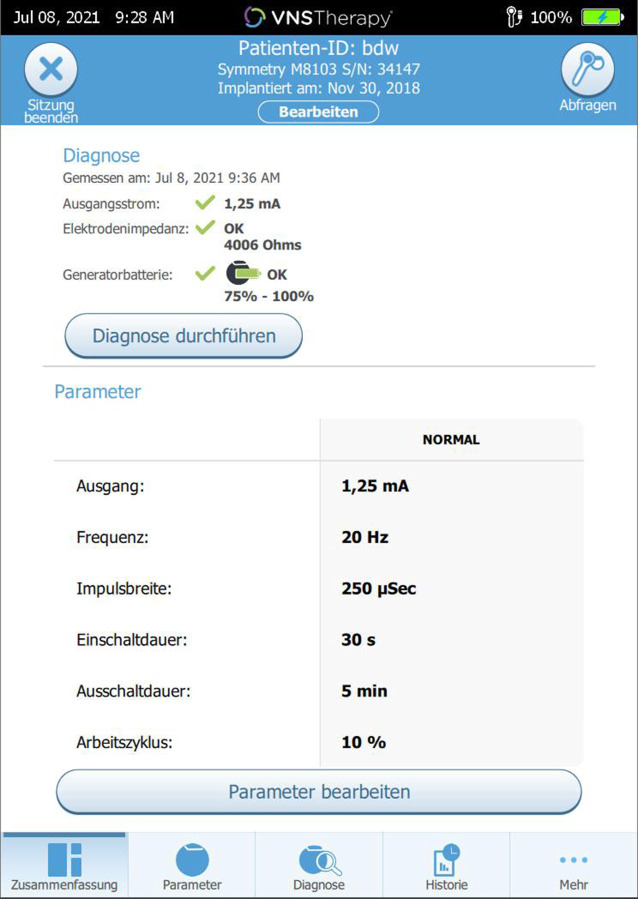

In einer ca. einstündigen Operation in Vollnarkose wird der Schrittmacher (Impulsgenerator) unterhalb der linken Klavikula positioniert. Von dort aus verläuft ein dünnes Kabel unter der Haut zum linken N. vagus (Abb. 1), welches um den Nerv gewunden wird und die Schrittmacherimpulse an den Nerven abgibt. Diese werden über die afferenten Fasern zum Hirnstamm und von dort zu subkortikalen Strukturen vorwiegend des limbischen Systems weitergeleitet (Abb. 2). Der Schrittmacher stimuliert in der Grundeinstellung alle 5 min für 30 s im sehr niedrigen Milliamperebereich mit einer bestimmten Impulsfrequenz und -breite; diese fünf Parameter können im Verlauf mittels der auf einem Tablet laufenden Software angepasst werden (Abb. 3).

Der N. vagus projiziert afferent zum neuromodulatorischen Netzwerk mit Beteiligung des Locus coeruleus und der dorsalen Raphe-Kerne (Abb. 2), also den Kerngebieten der serotonergen und noradrenergen Neurotransmission [1]. Dementsprechend führt die VNS im Mausmodell zu einer gesteigerten extrazellulären Noradrenalin- und Dopaminkonzentrationen in kortikalen (insbesondere hippokampalen) Regionen [2, 3]. Die VNS steigert außerdem die Serotoninrezeptordichte im Hippokampus von Mäusen, bei denen diese durch chronischen Stress vermindert war, und erhöht die Produktion des neurotrophen Faktors BNDF (Brain Derived Neutrophic Factor) [4]. Beobachtet wurde darüber hinaus eine Erhöhung der extrazellulären Serotoninkonzentration in den dorsalen Raphe-Kernen [3]. Die Wirkweise der VNS umfasst wahrscheinlich aber auch Effekte jenseits der monoaminergen Neurotransmission. Der N. vagus entfaltet antiinflammatorische Effekte sowohl durch zentrale Aktivierung antiinflammatorischer Pathways, wie die Aktivierung des Hypothalamus-Hypophysen-Nebennieren-Stoffwechsels, als auch durch die periphere parasympathische Inhibition der Produktion proinflammatorischer Zytokine wie TNF, IL(Interleukin)-1ß, IL‑6 und IL-18 [5]. Diese Inhibition der Zytokinproduktion konnte auch bei Autoimmunerkrankungen wie der rheumatoiden Arthritis [6] oder Morbus Crohn [7] gezeigt werden. Aufgrund dieser Befunde wurde diskutiert, ob eine Modulation der mit Depressionen assoziierten subklinischen Inflammation ein therapeutisches Wirkprinzip der VNS darstellt [8]. Auf der Systemebene konnte mittels FDG-PET gezeigt werden, dass im Verlauf der Therapie bei VNS-Respondern eine Reduktion des rechtshemisphärischen Glukosemetabolismus im Bereich des dorsolateralen präfrontalen Kortex (DLPFC) und eine Steigerung des linkshemisphärischen Glukosemetabolismus eintritt, sodass eine Regulation der bei der Depression beobachteten interhemisphärischen Dysbalance durch die VNS postuliert wurde [9]. Funktionelle Untersuchungen der Hirnaktivität zeigten, dass die *transkutane* VNS zur Pupillendilatation und zur Reduktion der α‑Oszillationen im EEG führt [10]. Unter Berücksichtigung der Tatsache, dass eine gesteigerte α‑Power mit depressiven Zuständen in Zusammenhang gebracht wurde [11] und invers zur kortikalen Aktivität korreliert [12], kann als Hypothese formuliert werden, dass die Reduktion der α‑Aktivität durch die VNS auf eine kortikale Aktivierung hindeutet, die möglicherweise noradrenerg über den Locus coeruleus vermittelt wird [10]. Wie bei anderen antidepressiven Therapieverfahren auch, scheint der Wirkmechanismus der VNS also heterogen zu sein.

## Klinische Befunde zur antidepressiven Wirksamkeit der VNS

Die invasive VNS ist seit 1994 in der Europäischen Union und seit 1997 in den USA für die Behandlung der medikamentös therapierefraktären Epilepsie des Kindesalters zugelassen [13]. Bei Erwachsenen zeigten sich positive Effekte auf die Stimmung nach ca. 3 Monaten VNS, die anhaltend und von der antikonvulsiven Effektivität unabhängig waren [14], sodass ein genuin antidepressiver Effekt der VNS postuliert wurde, was die Weiterentwicklung als antidepressives Therapieverfahren anstieß.

**Figure 4:**
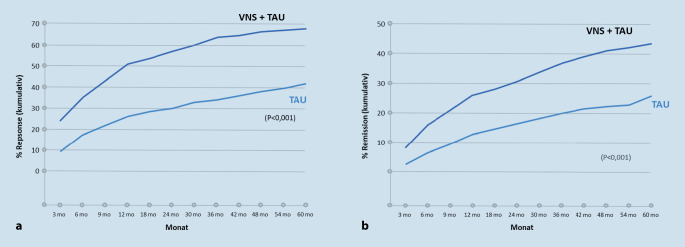

Im Jahr 2001 wurde einem VNS-System in der EU erstmals eine CE-Zertifizierung für die Behandlung der chronischen oder rezidivierenden Depression bei Patienten erteilt, die unter einer therapieresistenten Depression leiden oder von denen die aktuelle Depressionsbehandlung nicht toleriert wird [15]. 2005 wurde die VNS in den USA zur Behandlung der therapieresistenten Major-Depression bei Patienten über 18 Jahren zugelassen, die auf mindestens vier antidepressive Behandlungsstrategien nicht adäquat respondiert haben [16]. In die Zulassungsstudie wurden Patienten mit einer nichtpsychotischen, nichtatypischen uni- oder bipolaren Depression eingeschlossen [17]. In den letzten 20 Jahren gab es zahlreiche Studien und Fallserien zur VNS bei therapieresistenten depressiven Patienten. Viele unterstreichen den zusätzlichen Profit von VNS als adjuvantes Verfahren, sind allerdings vom Design her naturalistische Beobachtungsstudien; Sham-kontrollierte Studien fehlen weitgehend aufgrund methodischer Schwierigkeiten bei der Verblindung und ethischer Probleme bei der Verwendung von Sham-Bedingungen im Rahmen eines operativen Verfahrens. Die größte Langzeitstudie stammt von Aaronson und Mitarbeitern [18]. Diese Registerstudie untersuchte den klinischen Verlauf von 494 Patienten mit TRD mit „treatment as usual“ (TAU) plus VNS im Vergleich zu 301 Patienten mit lediglich TAU über 5 Jahre. Sowohl das kumulative Ansprechen auf die Therapie (68 % vs. 41 %) als auch die Remissionsraten (43 vs. 26 %) waren signifikant größer in der mit VNS behandelten Gruppe (Abb. 4). Patienten, die zuvor von mindestens einer Elektrokonvulsionstherapie(EKT)-Serie mit mindestens 7 Sitzungen profitiert hatten, zeigten ein besonders gutes Ansprechen auf die VNS-Behandlung, aber auch EKT-Nonresponder sprachen besser auf die kombinierte Behandlung an als auf TAU allein. Ebenfalls bedeutsam war, dass sowohl Response- als auch Remissionsrate über den Behandlungszeitraum kontinuierlich anstiegen. Bislang gibt es nur eine Sham-kontrollierte Studie zur VNS-Behandlung bei TRD [19]. Hier war die VNS einer Sham-Stimulation über einen Beobachtungszeitraum von 10 Wochen nicht signifikant überlegen. In der naturalistischen Nachverfolgung diese Patienten über 12 Monate stieg die Response der VNS-Patienten über die Zeit deutlich an. In weiteren langfristigen Beobachtungsstudien hat die VNS verglichen mit TAU einen höheren antidepressiven Effekt, der sich allerdings erst nach einer Behandlungsdauer von 12 Monaten oder mehr entfaltet [20]. Zusammengefasst deuten die Daten also darauf hin, dass Unterschiede in der Responserate und bei den Therapieeffekten erst im längerfristigen Verlauf nach 3 bis 12 Monaten zu beobachten sind und mit zunehmender Therapiedauer die VNS-Effekte auch größer werden, was darauf hindeutet, dass der Wirkmechanismus der VNS auf neuroplastische bzw. adaptive Phänomene zurückzuführen sein dürfte. Das negative Ergebnis der randomisierten kontrollierten Studie liegt also möglicherweise an der zu kurzen Beobachtungszeit und steht auch nicht in Widerspruch zu der o. g. Registerstudie, bei der die erste Studienvisite nach 3 Monaten stattfand.

### Vergleich internationaler Leitlinienempfehlungen

In der Nationalen Versorgungsleitlinie (NVL) „Unipolare Depression“ aus dem Jahr 2015 [21] wird die VNS neben der repetitiven transkraniellen Magnetstimulation (rTMS) bei den neueren nichtpharmakologischen therapeutischen Möglichkeiten aufgelistet. Die Leitlinie bezieht sich auf offene Studien, die eine Reduktion von Depressionssymptomen zeigen, und formuliert als Statement, dass zu wenig Evidenz insbesondere aus kontrollierten Studien existiert, um Empfehlungen für die allgemeine klinische Nützlichkeit und Anwendbarkeit aussprechen zu können. Die kanadische Leitlinie empfiehlt auf Basis der vorliegenden Befunde die VNS als Therapie der 3. Wahl für TRD nach repetitiver transkranieller Magnetstimulation (rTMS) und EKT, vergleichbar mit dem Empfehlungsgrad für die transkranielle Direktstromstimulation (tDCS; [22]). Mit Verweis auf die Langzeitwirkung nach 12 und 24 Monaten wird empfohlen, dass bei Patienten mit chronischer Depression VNS erwogen werden kann, insbesondere in Fällen, bei denen Probleme mit der Therapieadhärenz bestehen [22].

Die Leitlinie der American Psychiatric Association aus dem Jahr 2009 erwähnt die VNS als mögliche zusätzliche Behandlung bei Patienten, die im Vorfeld auf mindestens vier antidepressive Behandlungsversuche einschließlich EKT nicht respondiert haben. Die Leitlinie hebt den potenziellen Nutzen in der Langzeitbehandlung mit geringer, aber anhaltender Besserung hervor, während von der Anwendung zur alleinigen Akutbehandlung abgeraten wird [23]. Im Jahre 2020 hat das britische National Institute for Health and Care Excellence (NICE) ein Positionspapier zur Anwendung der VNS bei TRD veröffentlicht [24]. Hier wird festgehalten, dass trotz typischer Nebenwirkungen wie Stimmveränderungen, Husten und Luftnot, die mit der Zeit abnehmen, die Behandlung gut vertragen wird und keine schwerwiegenden Sicherheitsbedenken bestehen [24].

Insbesondere das Fehlen weiterer randomisierter kontrollierter Langzeitstudien führt zu den zurückhaltenden Leitlinienempfehlungen. Allerdings ist erwähnenswert, dass solche Studien zwar den Goldstandard der evidenzbasierten Medizin darstellen, bei solch invasiven Therapiemaßnahmen aber entsprechende ethische Diskussionen mit sich bringen. Möglicherweise könnten hier bessere Studiendesigns offener Studien (bspw. durch prospektives „propensity score matching“) ebenfalls zu höherqualitativer Evidenz führen.

## Klinische Durchführung der VNS

### Indikation und Patientenauswahl

Die VNS als ergänzende Therapie zur Standardbehandlung ist grundsätzlich bei Patienten mit der Hauptdiagnose einer depressiven Störung geeignet. Die Zulassung erfolgte für „chronische und rezidivierende Depressionen, die sich in einer therapieresistenten depressiven Episode befinden“. Hier stellt sich die Frage nach einer allgemeingültigen Definition von Therapieresistenz, die bislang nicht existiert [25]. Die europäische Zulassungsbehörde EMA nimmt Therapieresistenz an, wenn mindestens zwei Antidepressiva aus unterschiedlichen Substanzklassen in ausreichender Dauer und Dosis nicht zu einer signifikanten Verbesserung führten. Dabei zielen die Therapieversuche immer auf Remission und Akutbehandlungen ab, Rezidive und chronifizierende Verläufe werden im Konzept der Therapieresistenz nicht ausreichend mitgedacht. Die konzeptuelle Schwierigkeit der Therapieresistenz adressierend erschien kürzlich eine Konsensusveröffentlichung für das Konzept der „schwer zu behandelnde Depression“ („difficult to treat depression“; [26]). Dieses geht pragmatischer von schwer zu behandelnden Depressionen mit einem Modell der chronischen Erkrankung aus und sucht Wege der Symptomreduktion und Verbesserung der Lebensqualität für die schwerkranken Patienten. Hier werden im Gegensatz zum kategorialen Konzept der Therapieresistenz Verbesserung und Verschlechterung der Erkrankung in einem Kontinuum gesehen.

Im Falle einer chronischen depressiven Störung sollte die Dauer der depressiven Episode unserer Einschätzung nach mindestens ein Jahr (in manchen Zentren > 2 Jahre) betragen; bei einer rezidivierenden depressiven Episode (ICD-10 F33.2) sollte der Patient drei oder mehr Episoden (einschließlich der aktuellen) in den letzten 10 Jahren gehabt haben. Wichtig bei der Entscheidung ist die Beeinträchtigung von Lebensqualität und Teilhabe durch die Erkrankung, die sich in Residualsymptomen, häufigen Krankenhausaufenthalten oder hoher Last an medikamentöser Behandlung zeigen kann. Je mehr solche invalidierenden Faktoren hinzutreten, umso eher kann die Indikation zur VNS als Zusatzbehandlung gestellt werden. Ebenfalls für VNS geeignet sind Patienten mit einer schwer zu behandelnden bipolaren Depression, wobei hier das Kriterium der Therapieresistenz noch unschärfer definiert ist [27]. Bei Patienten mit bipolar-affektiven Störungen sollte der prädominante Pol die Depression sein. In seltenen Fällen wurde bei unipolaren Patienten über einen Wechsel in eine manische Episode unter VNS berichtet, wobei das Risiko des Auftretens nicht über dem naturalistischen Switch-Risiko lag (u. a. 19) und die manischen Episoden gut zu behandeln waren. Nach einer Fortführung der VNS trat keine weitere manische Phase auf.

Selbstverständlich sollten alle infrage kommenden Patienten bislang gemäß den gültigen Leitlinien behandelt worden sein. Die Patientenauswahl (Infobox 1) erfordert eine gute Kenntnis des bisherigen Krankheits- und Behandlungsverlaufs, der aktuellen Symptomatik und der Komorbiditäten des Patienten sowie letztlich eine Abwägung des Nutzens und der Risiken im individuellen Fall. Da für die Kostenübernahme durch die Krankenkassen eine Therapieresistenz nachgewiesen sein muss, sollte der Patient von *mindestens zwei Antidepressiva verschiedenen Wirkprinzips* nicht profitiert haben. Darunter sollte aus unserer Sicht mindestens **ein ***Medikament mit mindestens dualen Wirkmechanismus *(SNRI, TZA) wie z. B. Venlafaxin oder Amitriptylin gewesen sein. Ferner sollten mindestens zwei Augmentationsstrategien gemäß der NVL „Unipolare Depression“ eingesetzt worden sein, typischerweise Lithium und ein Antipsychotikum der 2. Generation (Quetiapin, Aripiprazol oder Risperidon).

Aus der bereits erwähnten Registerstudie von Aaronson [18] lässt sich eine Empfehlung für EKT-Responder ableiten, die nach einer erfolgreichen EKT-Serie wiedererkranken, die Behandlung nicht mehr vertragen oder in einem Erhaltungs-EKT-Schema verbleiben müssen, um eine Remission aufrecht zu erhalten. Die Responsewahrscheinlichkeit ist deutlich höher, wenn der Patient in der Vergangenheit positiv auf EKT angesprochen hat, sodass hier aus unserer Sicht frühzeitig VNS angeboten werden sollte, insbesondere wenn die Krankheitslast des Patienten entsprechend hoch ist. Bei Patienten in einem Erhaltungs-EKT-Schema bietet sich die Therapie oft geradezu an, da diese Patienten sich in aller Regel eine Erweiterung der Abstände zwischen den Erhaltungs-EKT wünschen. In einer Fallserie mit 10 Patienten, die mit Erhaltungs-EKT behandelt wurden, konnten 7 davon ein Jahr nach Beginn der zusätzlichen VNS auf eine Erhaltungs-EKT verzichten [28]. In Anbetracht der begrenzten therapeutischen Optionen bei EKT-Non- oder Partialrespondern kann VNS eine sinnvolle Langzeitbehandlung sein, auf die immerhin mehr als die Hälfte der Patienten respondieren [18]. Auch bei Patienten, die sehr unter Nebenwirkungen der Medikation leiden oder bei denen eine Pharmakotherapie (relativ) kontraindiziert ist, kann die VNS als Therapie erwogen werden.

In einer kleinen Beobachtungsstudie mit 19 Patienten konnte gezeigt werden, dass Patienten mit einer initial höheren QTc-Zeit im EKG eine größere Symptomverbesserung nach 12 Monaten zeigten, wenn sie mit VNS behandelt wurden, wobei QTc und Symptomverbesserung signifikant korrelierten [29]. Die Stichprobe war allerdings zu klein, um die QTc als prognostischen Biomarker für ein Therapieansprechen empfehlen zu können. In einer aktuellen Veröffentlichung wurden Vorschläge zur Auswahl von Patienten entwickelt, die im Wesentlichen die hier genannten Aspekte berücksichtigen, jedoch auf das britische Versorgungssystem zugeschnitten sind und deshalb nicht vollständig auf hiesige Gegebenheiten übertragen werden können [30].

Es gibt nur sehr wenig Daten, die eine VNS-Therapie bei Patienten mit Rapid-cycling-Verlauf einer bipolar-affektiven Störung unterstützen, daher raten wir von einem Einsatz bei dieser Verlaufsform ab. Kritisch muss die VNS bei Patienten betrachtet werden, die an anderen psychischen Erkrankungen als Depressionen leiden. Insbesondere für primär psychotische Erkrankungen gibt es kaum Hinweise auf eine Wirksamkeit der VNS. Das Vorliegen einer schweren Depression mit psychotischen Symptomen ist hingegen kein Ausschlussgrund für eine VNS-Behandlung. Zurückhaltung ist auch geboten bei Patienten, bei denen eine schwere Persönlichkeitsstörung besonders aus den Clustern A und B vorliegen, da sich diese u. a. erschwerend auf die Adhärenz auswirken können. Gleiches gilt für Patienten mit schweren Abhängigkeitserkrankungen. Außerdem müssen die Patienten aufklärungsfähig und in der Lage sein, die Tragweite des operativen Eingriffs und die Konsequenzen der Behandlung zu verstehen, und sollten auch bereit sein, die nötigen Folgetermine zur Dosistitration und zum Monitoring wahrzunehmen.

Die VNS-Stimulation kann ein bestehendes obstruktives Schlafapnoesyndrom (OSAS) verschlechtern, daher sind schwere Verläufe eines OSAS eine Kontraindikation, ebenso wie eine stattgehabte Vagotomie des linken N. vagus.

## Nebenwirkungen der VNS

Die Patienten müssen in der Aufklärung vor VNS über typische und häufige chirurgische Nebenwirkungen wie Schmerzen und Parästhesien informiert werden. Durch Irritation des Nervs bestehen bei etwa jedem 3. Patienten postoperativ Heiserkeit und Stimmveränderung, auch unabhängig von der Stimulation. Diese remittieren in der Regel innerhalb weniger Wochen. Ernste Nebenwirkungen und Komplikationen wie z. B. passagere Schluckstörungen infolge (partieller) Vagusnervparese sind selten [31].

Die häufigste mit der Stimulation assoziierte Nebenwirkung der VNS ist Heiserkeit, die bei etwa 60 % der Patienten auftritt und 12 Monaten postoperativ bei etwa der Hälfte der Patienten während der Stimulation noch bemerkt wird [32]. Weitere typische Nebenwirkungen im 12-Monats-Follow-up sind u. Dyspnoe (30 %), Schmerzen (28 %), Husten (26 %), Parästhesien (23 %), Kopfschmerzen (22 %), Dysphagie (16 %) und Schlafstörungen (11 %; [33]). Weitere Nebenwirkungen können Laryngismus, Hals- und Nackenschmerzen, Hypertonie, Nausea und Pharyngitis sein [31]. Durch Reduktion der Stimulationsintensität oder Senkung von Stimulationsfrequenz oder Impulsbreite können die stimulationsassoziierten Nebenwirkungen gemildert oder sogar beseitigt werden. Aufgrund von Kabelbrüchen oder zum Austausch der Batterie, die je nach Einstellung der Stimulationsparameter eine Lebensdauer von 3 bis 8 Jahren hat, kann ein erneuter kleiner chirurgischer Eingriff notwendig werden [31].

## Aufbau einer VNS-Ambulanz

Aufgrund der Schwere und Komplexität der Erkrankung und der notwendigen Behandlung sollten Patienten, bei denen eine VNS erwogen wird, in einem spezialisierten Zentrum mit einem multiprofessionellen Team behandelt werden. Folgende Aspekte sollten hierbei beachtet werden.

- Das psychiatrische Team sollte aus Fachärzten für Psychiatrie und Psychotherapie bestehen, die über Erfahrung in der Behandlung schwer zu behandelnder Depressionen verfügen. Es ist über die Auswahl der Patienten und die Implantation hinaus auch Ansprechpartner für den Patienten bei Dosistitration, Verlaufskontrollen, das Nebenwirkungsmanagement und in der Langzeittherapie und sollte daher sowohl in der VNS-Therapie erfahren als auch langfristig in der Behandlung involviert sein.
- Angehörige sollten frühzeitig bei der Indikationsstellung und der Aufklärung mit einbezogen werden. Dem Patienten und den Angehörigen sollten ausreichend Bedenkzeit und ggf. Folgetermine eingeräumt werden.
- Das Team sollte vom Hersteller des Implantats zur Fachinformation, zur Implantation, zu den Anwendungsbereichen, Sicherheitsaspekten, zur Nachsorge und zur Dosistitration geschult werden; ein persönlicher Ansprechpartner der Firma ist unerlässlich.
- Eine parallele Behandlung durch eine Hochschulambulanz und durch einen niedergelassenen Psychiater ist möglich. In diesem Fall ist eine enge Abstimmung prä- und postoperativ unabdingbar.
- Eine ambulante Psychotherapie sollte entsprechend den Leitlinien fortgeführt oder erneut begonnen werden.
- Eine lokale Kooperation mit einem neurochirurgischen Team, das über Erfahrung in der Implantation der VNS verfügt, ist wichtig. Es ist hilfreich, im psychiatrischen Gespräch auf die Expertise und Erfahrung des Neurochirurgen verweisen zu können, um Ängste der Patienten abzubauen. Möglicherweise können Neurochirurgen, die bislang VNS im Rahmen der Epilepsiebehandlung durchführen, die sich im Hinblick auf die chirurgischen Abläufe nicht von der „antidepressiven“ VNS unterscheidet, auch die Implantation bei Depressionspatienten übernehmen.
- Das neurochirurgische Team sollte möglichst aus mehreren Experten bestehen, um bei Abwesenheiten die Kontinuität der Versorgung sicherzustellen.
- Nachdem die psychiatrische Indikation für die VNS gestellt und mit dem Patienten und seinen Angehörigen diskutiert wurde, sollte ein Aufklärungsgespräch mit dem Neurochirurgen organisiert werden.

## Monitoring und Begleitung der Patienten

Laut der Fachinformation sollte die VNS-Stimulation ca. 14 Tage nach der Operation begonnen werden. Unserer Erfahrung nach ist es jedoch auch möglich, bereits wenige Tage postoperativ mit der ersten Einstellung zu beginnen. Hierdurch kann möglicherweise die Wirklatenz verkürzt werden [34]. Die Schnelligkeit der Aufdosierung der Stromstärke bis in den Zielbereich (ca. 1,5–1,75 mA) muss individuell je nach der Toleranz des Patienten gegenüber zunächst auftretenden und binnen Tagen verschwindenden unangenehmen Empfindungen bis hin zu Schmerz bei der Stimulation gehandhabt werden. Während bei einigen Patienten schon 0,25 mA pro Sitzung deutliche Nebenwirkung verursachen können, ist es bei anderen Patienten möglich bis zu 3 Schritte (insgesamt 0,75 mA) in einer Sitzung zu erhöhen. Titrationsvisiten können in mehrtägigem bis mehrwöchigem Abstand erfolgen, in der Regel sind 4 bis 6 Visiten erforderlich. Bei starken Nebenwirkungen kann es erforderlich sein, die Titration herabzusenken und einige Tage später die Erhöhung erneut zu versuchen. Ferner werden Patienten instruiert, mit einem Magneten, der ihnen mitgegeben wird, die Stimulation bei Bedarf (z. B. starken Nebenwirkungen oder beim Singen) auszuschalten. Nach Erreichen der Zielparameter (vgl. Abb. 3) wird der Patient in der Regel alle 3 Monate gesehen. Neben der Kontrolle und ggf. Anpassung der Stimulation erfolgen eine psychiatrische Untersuchung und ein psychometrisches Monitoring mit etablierten Ratingskalen wie z. B. MADRS, QIDS-16, oder BDI‑I bzw. -II. Die Nebenwirkungen werden ebenfalls bei jeder Sitzung abgefragt. Geräteeinstellung wie z. B. die Impedanz werden automatisch überprüft. Nach dem Erreichen des Zielbereichs für die Stimulationsstärke kann nach mehreren Monaten eine Änderung des Arbeitszyklus erwogen werden, um die Wirksamkeit weiter zu steigern. Hierbei erfolgt eine Reduktion der Auszeit des Stimulators, sodass die Stimulation insgesamt häufiger erfolgt und somit insgesamt eine höhere Stimulationsdauer erreicht wird.

## Ausblick und Diskussion

Die VNS bietet für Patienten mit schwer zu behandelnder Depression eine zugelassene Therapieoption, die als langfristige Zusatzbehandlung zur Erhöhung der Response- und Remissionswahrscheinlichkeit beitragen und zu einer Verbesserung der Lebensqualität führen kann [18, 35]. Offene Beobachtungsstudien haben entsprechende Wirksamkeitsnachweise erbracht [18, 32], das Fehlen positiver randomisierter kontrollierter Studien führt allerdings bislang zu eher zurückhaltenden Leitlinienempfehlungen. Weitere Studien sind nötig, um stärkere Evidenz für die Wirksamkeit zu generieren und auch den Wirkmechanismus besser zu verstehen. Akutbehandlungen depressiver Episoden mit EKT oder (Es‑)Ketaminpräparaten sind mit Problemen wie Residualsymptomen, Rückfallrisiko oder der Notwendigkeit häufiger Erhaltungstherapien behaftet, die durch die VNS als langfristige Therapie adressiert werden können. Auch Patienten, die beispielsweise Lithium oder andere Präparate zur Phasenprophylaxe einnehmen und möglicherweise unter nichttolerablen Nebenwirkungen leiden oder Kontraindikationen aufweisen, können von einer zusätzlichen Behandlung mit VNS profitieren, um beispielsweise Medikation einzusparen. Die Reduktion der Notwendigkeit von Erhaltungstherapien wurde für die EKT in einer kleinen Stichprobe nachgewiesen [28]. Für die anderen genannten Einsatzbereiche muss die Umsetzbarkeit noch in Studien gezeigt werden. Weitere Prädiktoren für eine Response sind wichtig, um die Patienten besser beraten zu können. Diese werden gerade in einer noch rekrutierenden Beobachtungsstudie [15] identifiziert. Patienten mit schwer zu behandelnden Depressionen benötigen in aller Regel mehrere oder sogar viele Therapiebausteine, um eine Verbesserung der Lebensqualität zu erreichen. Daher sollte VNS als nichtpharmakologische Therapiemethode unseres Erachtens als therapeutische Option mit Langzeiteffekt einbezogen werden, um diesen schwerkranken Patienten überdauernd helfen zu können.

### Infobox 1 Pragmatische Indikationskriterien für eine VNS-Therapie bei schwer zu behandelnder Depression

- Notwendige Voraussetzungen
  - Primäre Diagnose einer schweren depressiven Episode oder einer bipolar-affektiven Störung, aktuell schwere depressive Episode, die leitliniengemäß behandelt wird
    *und*
  - Dauer der aktuellen depressiven Episode > 1 Jahr (in manchen Zentren > 2 Jahre) *oder* ≥ 3 Episoden in den letzten ca. 10 Jahren
- Kriterien, wann eine VNS in Erwägung gezogen werden sollte
  - Unzureichendes Ansprechen auf mindestens zwei Antidepressiva unterschiedlicher Substanzklassen (idealerweise einschließlich eines Trizyklikums) in ausreichender Dosierung (TDM-kontrolliert) und Dauer sowie zwei Augmentationsstrategien (bspw. Lithium, Quetiapin) in Kombination mit einer Richtlinien-Psychotherapie
  - Nichttolerierbare Nebenwirkungen einer Pharmakotherapie bzw. Kontraindikationen gegen medikamentöse Therapieverfahren
  - EKT-Responder mit Rückfällen oder Residualsymptomen nach Beendigung der (Erhaltungs‑)EKT, nichttolerablen EKT-Nebenwirkungen oder der Notwendigkeit von Erhaltungs-EKT
  - Wiederholte oder lange Krankenhausbehandlungen aufgrund einer Depression
- Höhere Wahrscheinlichkeit eines Ansprechens auf VNS
  - Gute EKT-Response mit der Notwendigkeit einer Erhaltungs-EKT (↑↑↑)
  - Generell gute EKT-Response (↑↑)
  - Bei bipolar-affektiver Störung: prädominant depressive Polarität (↑)
- Niedrigere Wahrscheinlichkeit eines Ansprechens auf VNS
  - Relevante Persönlichkeitsstörung (↓↓)
  - Aktuelle Abhängigkeitserkrankung (↓↓)
  - Schizophrenie oder schizoaffektive Störung (↓)
  - Fragliche Compliance hinsichtlich der notwendigen Kontrolluntersuchungen (↓)

## Supplementary Information





## References

[1] Hulsey DR, Shedd CM, Sarker SF, Kilgard MP, Hays SA (2019). Norepinephrine and serotonin are required for vagus nerve stimulation directed cortical plasticity. Exp Neurol.

[2] Roosevelt RW, Smith DC, Clough RW, Jensen RA, Browning RA (2006). Increased extracellular concentrations of norepinephrine in cortex and hippocampus following vagus nerve stimulation in the rat. Brain Res.

[3] Manta S, El Mansari M, Debonnel G, Blier P (2013). Electrophysiological and neurochemical effects of long-term vagus nerve stimulation on the rat monoaminergic systems. Int J Neuropsychopharmacol.

[4] Shin HC, Jo BG, Lee CY, Lee KW, Namgung U (2019). Hippocampal activation of 5-HT1B receptors and BDNF production by vagus nerve stimulation in rats under chronic restraint stress. Eur J Neurosci.

[5] Borovikova LV, Ivanova S, Zhang M, Yang H, Botchkina GI, Watkins LR (2000). Vagus nerve stimulation attenuates the systemic inflammatory response to endotoxin. Nature.

[6] Koopman FA, Chavan SS, Miljko S, Grazio S, Sokolovic S, Schuurman PR (2016). Vagus nerve stimulation inhibits cytokine production and attenuates disease severity in rheumatoid arthritis. Proc Natl Acad Sci U S A.

[7] Bonaz B, Sinniger V, Hoffmann D, Clarençon D, Mathieu N, Dantzer C (2016). Chronic vagus nerve stimulation in Crohn’s disease: a 6-month follow-up pilot study. Neurogastroenterol Motil.

[8] Corcoran C, Connor TJ, O’Keane V, Garland MR (2005). The effects of vagus nerve stimulation on pro- and anti-inflammatory cytokines in humans: a preliminary report. Neuroimmunomodulation.

[9] Conway CR, Chibnall JT, Gebara MA, Price JL, Snyder AZ, Mintun MA (2013). Association of cerebral metabolic activity changes with vagus nerve stimulation antidepressant response in treatment-resistant depression. Brain Stimul.

[10] Sharon O, Fahoum F, Nir Y (2021). Transcutaneous vagus nerve stimulation in humans induces pupil dilation and attenuates alpha oscillations. J Neurosci.

[11] Leuchter AF, Cook IA, Jin Y, Phillips B (2013). The relationship between brain oscillatory activity and therapeutic effectiveness of transcranial magnetic stimulation in the treatment of major depressive disorder. Front Hum Neurosci.

[12] Fernández-Palleiro P, Rivera-Baltanás T, Rodrigues-Amorim D, Fernández-Gil S, del Carmen Vallejo-Curto M, Álvarez-Ariza M (2020). Brainwaves oscillations as a potential biomarker for major depression disorder risk. Clin EEG Neurosci.

[13] Toffa DH, Touma L, El Meskine T, Bouthillier A, Nguyen DK (2020). Learnings from 30 years of reported efficacy and safety of vagus nerve stimulation (VNS) for epilepsy treatment: a critical review. Seizure.

[14] Elger G, Hoppe C, Falkai P, Rush AJ, Elger CE (2000). Vagus nerve stimulation is associated with mood improvements in epilepsy patients. Epilepsy Res.

[15] Young AH, Juruena MF, De Zwaef R, Demyttenaere K (2020). Vagus nerve stimulation as adjunctive therapy in patients with difficult-to-treat depression (restore-life): study protocol design and rationale of a real-world post-market study. BMC Psychiatry.

[16] Johnson RL, Wilson CG (2018). A review of vagus nerve stimulation as a therapeutic intervention. J Inflam Res.

[17] Sackeim HA, Rush AJ, George MS, Marangell LB, Husain MM, Nahas Z (2001). Vagus nerve stimulation (VNS^TM^) for treatment-resistant depression: efficacy, side effects, and predictors of outcome. Neuropsychopharmacology.

[18] Aaronson ST, Sears P, Ruvuna F, Bunker M, Conway CR, Dougherty DD (2017). A 5-year observational study of patients with treatment-resistant depression treated with vagus nerve stimulation or treatment as usual: comparison of response, remission, and suicidality. Am J Psychiatry.

[19] Rush AJ, Marangell LB, Sackeim HA, George MS, Brannan SK, Davis SM (2005). Vagus nerve stimulation for treatment-resistant depression: a randomized, controlled acute phase trial. Biol Psychiatry.

[20] Sackeim HA, Dibué M, Bunker MT, Rush AJ (2020). The long and winding road of vagus nerve stimulation: challenges in developing an intervention for difficult-to-treat mood disorders. Neuropsychiatr Dis Treat.

[21] DGPPN, BÄK, KBV, AWMF (2015). S3-Leitlinie/Nationale VersorgungsLeitlinie Unipolare Depression – Langfassung.

[22] Milev RV, Giacobbe P, Kennedy SH, Blumberger DM, Daskalakis ZJ, Downar J (2016). Canadian Network for Mood and Anxiety Treatments (CANMAT) 2016 clinical guidelines for the management of adults with major depressive disorder: Section 4. Neurostimulation treatments. Can J Psychiatry.

[23] Gelenberg AJ, Freeman MP, Markowitz JC, Rosenbaum JF, Thase ME, Trivedi MH et al (2010) Practice guideline for the treatment of patients with major depressive disorder third edition work group on major depressive disorder. http://www.psychiatryonline.com/pracGuide/pracGuideTopic_7.aspx. Zugegriffen: 12.12.2021

[24] National Institute for Health and Care Excellence (2020) Implanted vagus nerve stimulation for treatment-resistant depression (IPG679). https://www.nice.org.uk/guidance/ipg679. Zugegriffen: 12.12.2021

[25] Brown S, Rittenbach K, Cheung S, McKean G, MacMaster FP, Clement F (2019). Current and common definitions of treatment-resistant depression: findings from a systematic review and qualitative interviews. Can J Psychiatry.

[26] Rush AJ, Aaronson ST, Demyttenaere K (2019). Difficult-to-treat depression: a clinical and research roadmap for when remission is elusive. Aust NZ J Psychiatry.

[27] McAllister-Williams RH, Sousa S, Kumar A, Greco T, Bunker MT, Aaronson ST (2020). The effects of vagus nerve stimulation on the course and outcomes of patients with bipolar disorder in a treatment-resistant depressive episode: a 5-year prospective registry. Int J Bipolar Disord.

[28] Aaronson ST, Goldwaser EL, Kutzer DJ, McAllister-Williams RH, Sackeim HA, Rush AJ (2020). Vagus nerve stimulation in patients receiving maintenance therapy with electroconvulsive therapy: a series of 10 cases. J ECT.

[29] Longpré-Poirier C, Desbeaumes Jodoin V, Miron JP, Fournier-Gosselin MP, Lespérance P (2020). Electrocardiogram corrected Q-T interval predicts response to vagus nerve stimulation in depression. J ECT.

[30] McAllister-Williams RH, Bulmer S, Newton K, Heath K, Cousins DA, Currie A (2021). Assessment for vagus nerve stimulation in patients with difficult-to-treat depression: a model from the Newcastle Regional Affective Disorders Service (RADS). J Affect Disord.

[31] LivaNova (2020) VNS Therapy® system depression physician’s manual. For healthcare professionals. https://dynamic.cyberonics.com/manuals/index_ifram. Zugegriffen: 12.12.2021

[32] Rush AJ, Sackeim HA, Marangell LB, George MS, Brannan SK, Davis SM (2005). Effects of 12 months of vagus nerve stimulation in treatment-resistant depression: a naturalistic study. Biol Psychiatry.

[33] Berry SM, Broglio K, Bunker M, Jayewardene A, Olin B, Rush AJ (2013). A patient-level meta-analysis of studies evaluating vagus nerve stimulation therapy for treatment-resistant depression. Med Devices Evid Res.

[34] Moeller S, Wang R, Aydin M, Lam AP, Sitter A, Grüter J (2020). Rapid titration protocol—experiences with a dynamic novel titration regime for vagus nerve stimulation in a group of depressive patients. J Clin Neurosci.

[35] Conway CR, Kumar A, Xiong W, Bunker M, Aaronson ST, Rush AJ (2018). Chronic vagus nerve stimulation significantly improves quality of life in treatment-resistant major depression. J Clin Psychiatry.

