# Supplementary material for: Vagus nerve stimulation for difficult to treat depression
Source: Nervenarzt. 2022 Apr 5;93(9):921–30. [Article in German] doi: 10.1007/s00115-022-01282-6 (PMC9452433; doi:10.1007/s00115-022-01282-6)
Supplement: Supplementary file 1 [file 115_2022_1282_MOESM1_ESM.docx]

**Online-Infobox 1: Kasuistiken**

**Kasuistik 1**

Eine 56-jährige Patientin mit rezidivierender Depression wurde aufgrund eines schweren depressiven Syndroms und einer suizidalen Krise notfallmäßig aufgenommen. Auf mehrere antidepressive Behandlungen respondierte die Patientin nicht. EKT, Lithium und Mirtazapin wurden aus Sorge vor Nebenwirkungen abgelehnt. Wir führten eine *off-label*-Therapie mit intravenösem Esketamin 0,5 mg/kg durch. Außerdem wurden Fluoxetin und Quetiapin ret. angesetzt. Nach 8 Infusionen kam es zu einer deutlichen Besserung des depressiven Syndroms. Parallel wurde sie aufgrund des langjährigen rezidivierenden Verlaufs mit VNS behandelt (1.5 mA, off-time 3 min.). Bei ausreichender Stabilität konnte die Esketamin-Intervalltherapie ca. 12 Monate nach Implantation der VNS beendet werden. Die Patientin begann eine berufsbildende Maßnahme. Sowohl Esketamin als auch VNS wurden gut vertragen.

**Kasuistik 2**

Die 1960 geborene Patientin beschreibt schon seit Jugendzeiten an immer wieder depressive Phasen. Die Erstdiagnose einer depressiven Störung erfolgte i.R. des ersten stationär-psychiatrischen Aufenthalts 1988. Seitdem gab es mindestens 8 stationär-psychiatrische Aufenthalte sowie sieben Suizidversuche. Zahlreiche medikamentöse antidepressive Vorbehandlungen sowie Augmentationsversuche mit Lithium, Quetiapin und L-Thyroxin blieben ohne überdauernde Stabilität. Die Patientin berichtete von zwei abgeschlossenen Richtlinienpsychotherapien (unterschiedliche Verfahren). Bereits in der Vergangenheit hatte sie gut von einer EKT-Serie profitiert.

Erster stat.-psychiatrischer Aufenthalt bei uns 2018 mit Partialremission unter EKT, danach Angebot und Implantation der VNS. Die Zielstimulationsparameter wurden innerhalb von fünf Wochen nach der Implantation erreicht, keine wesentlichen Nebenwirkungen der Therapie. Die Patientin beschreibt eine Verbesserung von Grundstimmung und Antrieb nach 5 Monaten, einige Monate später auch Verbesserung von Lebensfreude und -qualität. Die bestehende Medikation mit Clomipramin ret. und Lithium wurde unverändert belassen.
